# Tick-borne Agents in Rodents, China, 2004–2006

**DOI:** 10.3201/eid1512.081141

**Published:** 2009-12

**Authors:** Lin Zhan, Wu-Chun Cao, Chen-Yi Chu, Bao-Gui Jiang, Fang Zhang, Wei Liu, J. Stephen Dumler, Xiao-Ming Wu, Shu-Qing Zuo, Pan-He Zhang, Hai-Nan Huang, Qiu-Min Zhao, Na Jia, Hong Yang, Jan H. Richardus, J. Dik F. Habbema

**Affiliations:** Beijing Institute of Microbiology and Epidemiology, Beijing, People’s Republic of China (L. Zhan, W.-C. Cao, C.-Y. Chu, B.-G. Jiang, F. Zhang, W. Liu, X.-M. Wu, S.-Q. Zuo, P.-H. Zhang, H.-N. Huang, Q.-M. Zhao, N. Jia, H. Yang); Johns Hopkins University School of Medicine, Baltimore, Maryland, USA (J.S. Dumler); University Medical Center, Rotterdam, the Netherlands (J.H. Richardus, J.D.F. Habbema); 1 These authors contributed equally to this article.

**Keywords:** Anaplasma phagocytophilum, Borrelia burgdorferi sensu lato, spotted fever group rickettsiae, Francisella tularensis, rodents, coexistence, China, research

## Abstract

A total of 705 rodents from 6 provinces and autonomous regions of mainland People’s Republic of China were tested by PCRs for tick-borne agents (*Anaplasma phagocytophilum*, *Borrelia burgdorferi* sensu lato, spotted fever group rickettsiae, and *Francisella tularensis*). Infection rates were 5.5%, 6.7%, 9.1% and 5.0%, respectively. Eighteen (2.6%) rodents of 10 species were positive for 2 or 3 agents. Sequence analysis of PCR products confirmed the presence and genotypes of detected agents. These findings demonstrate that these tick-borne agents cocirculate and that a variety of rodent species may be involved in their enzootic maintenance.

## Abstract 2

Many rodent species may be involved in the enzootic maintenance of these agents.

*Anaplasma phagocytophilum*, *Borrelia burgdorferi* sensu lato, spotted fever group (SFG) rickettsiae, and *Francisella tularensis* are the causative agents of human granulocytic anaplasmosis, Lyme disease, spotted fever, and tularemia, respectively. These agents are naturally maintained in animal reservoirs and considered emerging or reemerging pathogens with serious public health implications. Although these agents could infect humans through various routes, ticks play a major role in transmission from animal hosts to humans.

Co-infection with these agents has been found in many tick species including *Ixodes scapularis* in northeastern United States, *I*. *pacificus* and *I*. *spinipalpis* in the western United States *I*. *ricinus* in Europe, and *I*. *persulcatus* in Asia (*1*). Patients co-infected with 2 tick-borne pathogens usually show more severe clinical signs of longer duration (*1*). Experimental concurrent infections with *A*. *phagocytophilum* and *B*. *burgdorferi* may suppress interleukin-2 (IL-2) and interferon-γ production, promote IL-4 response, increase pathogen load, and intensify Lyme arthritis (*2**–**4*). Natural infection and co-infection with these 4 agents have been reported in the People’s Republic of China in various tick species (*5**–**7*) such as *I*. *persulcatus*, *Dermacentor silvarum*, *Haemaphysalis concinna*, *H*. *longicornis*, and *H*. *warburconi*, which are known to feed on small mammals as well as humans.

We hypothesize that multiple agents might be present in rodents from tick-infested areas. The purpose of this study was to identify *A*. *phagocytophilum*, *B*. *burgdorferi*, SFG rickettsiae, and *F*. *tularensis* in rodents from mainland China and to better understand the public health role of these emerging and reemerging pathogens.

## Materials and Methods

### Sample Collection

During 2004–2006, rodents were collected at 6 study sites in Heilongjiang Province, Inner Mongolia Autonomous Region, Jilin Province, Zhejiang Province, Guizhou Province, and Xinjiang Autonomous Region (Figure) at various times according to peak seasons of tick species. The first 3 sites were forested highlands in the Small Xing’an Mountains and the Great Xing’an Mountains of northeastern China, where local residents worked and were exposed to rodents and ticks. The study sites in Zhejiang and Guizhou provinces were forested rolling hills with typical temperate zone vegetation; these regions attract hundreds of thousands of tourists per year. The study site in Xinjiang Autonomous Region was a forest with a rural resident population. Rodents were trapped by using peanuts as bait. After captured rodent species were identified, spleen specimens were collected and stored at –20°C until DNA was extracted.

**Figure:**
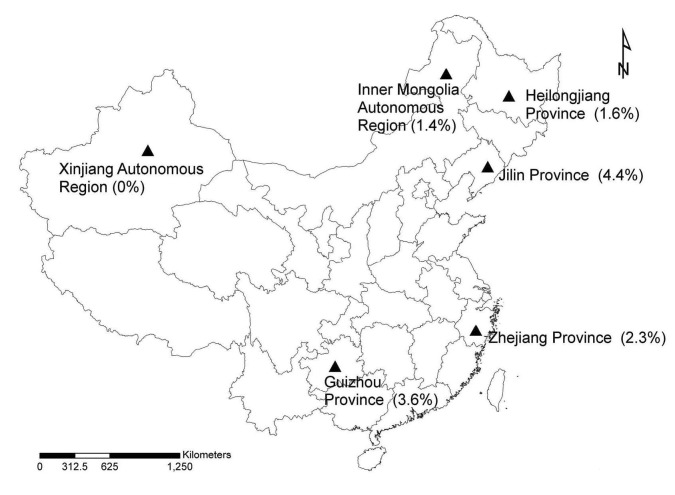
Study sites (triangles) in the People’s Republic of China where rodents were collected, 2004–2006. Numbers in parentheses are co-infection rates of rodents with 2 or 3 tick-borne agents.

### Extraction of DNA

Total DNA was extracted from spleen samples by using Trizol agent (Invitrogen, Carlsbad, CA, USA) following the instructions of the manufacturer. Briefly, ≈300 mg of spleen tissue from each rodent was crushed in Trizol reagent, and DNA was separated from RNA by centrifugation. DNA was precipitated after washing twice in a solution containing 0.1 M sodium citrate in 10% ethanol. The DNA pellet was then washed in 75% ethanol and kept at room temperature for 10–20 min. After centrifugation at 2,000 × *g* at 2–8°C for 5 min, DNA was dissolved in 8 mmol/L NaOH and centrifuged to remove insoluble material. The supernatant containing DNA was removed and adjusted with HEPES buffer to a pH of 7–8.

### PCR

Nested PCR was conducted with primers designed to amplify part of the 16S rRNA gene of *A*. *phagocytophilum*, as described (*8*). For amplification of *B*. *burgdorferi* DNA, a nested PCR was performed with primers derived from *B*. *burgdorferi* 5S–23S rRNA intergenic spacer (*9*). PCR was performed by using primers Rr 190.70p and Rr 190–701n to amplify a fragment of the gene encoding a 190-kDa outer membrane protein A (*ompA*) gene specific for SFG rickettsiae (*10*). Samples were tested for *F*. *tularensis* by a nested PCR specific for the outer membrane protein (*fopA*) gene, as described (*11*). All PCRs were performed by using a model 2700 thermal cycler (Perkin-Elmer, Waltham, MA, USA). PCR products were separated by agarose gel electrophoresis, stained with ethidium bromide, and examined under UV light. To avoid contamination, we performed DNA extraction, reagent setup, amplification, and agarose gel electrophoresis in separate rooms and included negative controls (distilled water) were in each amplification.

### DNA Sequencing and Analysis

PCR products of positive samples were sequenced directly by using a dideoxynucleotide cycle sequencing method with an automated DNA sequencer (ABI PRISM 377; Perkin-Elmer). To limit errors in sequencing, we performed 2 sequencing reactions of each PCR product. When different sequences were obtained, additional sequencing reactions were conducted to generate a consensus sequence. Sequences obtained in the present study were compared with the corresponding sequences deposited in GenBank by using the BLAST program of the National Center for Biotechnology Information (http://blast.ncbi.nlm.nih.gov/Blast.cgi).

### Statistical Analysis

Chi-square or Fisher exact tests were used to compare proportions. p values <0.05 were considered statistically significant.

## Results

A total of 705 rodents were captured. The number of rodents tested and infectivity rates at different survey sites are shown in the Table. *A*. *phagocytophilum* was detected only in rodents captured in eastern regions of China (Heilongjiang, Jilin, and Zhejiang provinces) (Figure). *B*. *burgdorferi* was detected in rodents captured at all 6 survey sites. SFG rickettsiae were detected in rodents captured at all sites except Jilin Province. *F*. *tularensis* was detected in rodents captured only in northern China (Heilongjiang and Jilin provinces and Inner Mongolia and Xinjiang autonomous regions; Figure).

**Table:** Infection rates for 4 tick-borne agents in rodents, People’s Republic of China, 2004–2006*

Study site	No. rodents tested	No. (%) rodents positive				p value
		*Anaplasma phagocytophilum*	*Borrelia burgdorferi*	SFG rickettsiae	*Francisella tularenesis*	
Heilongjiang Province	64	3 (4.7)	3 (4.7)	1 (1.6)	5 (7.8)	0.424
Jilin Province	205	20 (9.8)	17 (8.3)	0	26 (12.7)	0.329
IMAR	148	0	8 (5.4)	32 (21.6)	2 (1.4)	0.0001
XJAR	44	0	1 (2.3)	4 (9.1)	2 (4.5)	0.348
Zhejiang Province	216	16 (7.4)	16 (7.4)	21 (9.7)	0	0.598
Guizhou Province	28	0	2 (7.1)	6 (21.4)	0	0.252
Total	705	39 (5.5)	47 (6.7)	64 (9.1)	35 (5.0)	

*SFG, spotted fever group; IMAR, Inner Mongolia Autonomous Region; XJAR, Xinjiang Autonomous Region.

In Heilongjiang Province, all 4 agents were detected in rodents at similar frequencies (χ^2^ 2.80, df 3, p = 0.424). No SFG rickettsiae were detected in rodents from Jilin Province. The infectivity rates for the 3 agents in Jilin Province did not significantly differ (χ^2^ 2.23, df 2, p = 0.328). Infectivity rates for SFG rickettsiae were significantly higher than those for *B*. *burgdorferi* and *F*. *tularensis* in rodents from Inner Mongolia Autonomous Region (χ^2^ 39.76, df 2, p<0.001). Infectivity rates for the 3 agents in Xinjiang Autonomous Region did not differ significantly (χ^2^ 5.01, df 2, p = 0.082). Except for *F*. *tularensis*, the other 3 agents showed similar infectivity rates for Zhejiang Province (χ^2^ 1.30, df 2, p = 0.523). Only *B*. *burgdorferi* and SFG rickettsiae were found in Guizhou Province, and the difference in their infectivity rates was not significant (p = 0.525, by Fisher exact test).

A total of 18 (2.6%, 95% confidence interval 1.4%–3.8%) rodents from all survey sites except Xinjiang Autonomous Region were positive for 2 or 3 agents, among which 15 were positive for 2 agents. A *Clethrionomys rufocanus* rodent from Heilongjiang Province was positive for *A*. *phagocytophilum*, *B*. *burgdorferi*, and SFG rickettsiae, and 2 rodents (*Apodemus agrarius* and *Tamias sibiricu*) from Jilin Province were positive for *A*. *phagocytophilum*, *B*. *burgdorferi*, and *F*. *tularensis* (Appendix Table 1).

Overall, except for 6 unclassified rodents, 23 species of rodents captured at the 6 survey sites were identified. Rodent species composition varied greatly at different sites ( Appendix Table 2). *Rattus norvegicus* rodents were found at all survey sites except Xinjiang Autonomous Region. *A*. *agrarius*, *A*. *peninsulae*, *Clethrionomys rufocanus*, *Mus musculus*, and *T*. *sibiricu* rodents were found in northeastern China; *A*. *sylvaticus*, *Niviventer confucianus*, and *R*. *losea* were found mainly in southern China; and *Meriones unguieulataus* and *M*. *musculus* were found mainly in western China.

The dominant rodent species differed at various study sites. *C*. *rufocanus* (57.8%) was dominant in Heilongjiang Province, *A*. *agrarius* (36.2%) and *A*. *peninsulae* (27.1%) in Jilin Province, *A*. *agrarius* (29.7%) and *Microtus maximowiczii* (23.7%) in Inner Mongolia Autonomous Region, *M*. *musculus* (50.0%) and *M*. *unguieulataus* (34.1%) in Xinjiang Autonomous Region, *N*. *confucianus* (53,0%) in Zhejiang Province, and *R*. *norvegicus* (32.14%) and *M*. *musculus* (28.6%) in Guizhou Province ( Appendix Table 2).

To confirm the presence and determine genotypes of detected organisms, PCR products were sequenced and analyzed. A 919-bp partial 16S rRNA gene fragment for *A*. *phagocytophilum* was obtained from each positive specimen (*8*). *A*. *phagocytophilum* sequences detected in rodents from Heilongjiang and Jilin provinces (GenBank accession no. DQ342324) were identical and differed from those from Zhejiang Province (GenBank accession no. DQ458808) by 2 bp, from those from ticks in United Kingdom and Sweden (GenBank accession nos. AY082656 and AJ242784.1, respectively) by 2 bp, and from other known *A*. *phagocytophilum* sequences by >3 bp.

Sequence analysis of the *B*. *burgdorferi* 5S–23S rRNA intergenic spacer showed that agents isolated from rodents in Heilongjiang Province, Inner Mongolia Autonomous Region, Jilin Province, and Xinjiang Autonomous Region belonged to the *B*. *garinii* genospecies, similar to agents detected in ticks (GenBank accession no. DQ150540) in northern China. Of 16 *B*. *burgdorferi* detected in Zhejiang Province, 12 belonged to the *B*. *garinii* genospecies and the other 4 belonged to the *B*. *valaisiana*–related group (GenBank accession nos. EU160458 and EU160459). The 2 strains found in Guizhou Province also belonged to the *B*. *valaisiana*–related group (GenBank accession no. EU247840).

For identification of SFG rickettsiae, partial nucleotide sequences of the *ompA* gene were obtained from positive specimens in Heilongjiang Province and Inner Mongolia Autonomous Region. All sequences were identical to those of the *R*. *sibirica* genotype (GenBank accession no. U43807). Nucleotide sequences of 35 specimens positive for *F*. *tularensis* were identical to each other and to published sequences for the *F*. *tularensis* subsp. *holarctica* strain (GenBank accession no. AF247642.2).

## Discussion

We detected *A*. *phagocytophilum*, *B*. *burgdorferi*, SFG rickettsiae, and *F*. *tularensis* in diverse species of rodents from different areas of China. Our findings and previous evidence (*6**,**9**,**12**–**15*) suggest that several tick-borne agents cocirculate in mainland China, and a variety of rodent species may be involved in enzootic maintenance of these agents.

This study was not intended to be a comprehensive survey on active infections with *A*. *phagocytophilum*, *B*. *burgdorferi*, SFG rickettsiae, and *F*. *tularensis*. Rather, it was designed to investigate the presence and extent of these agents in China. If one considers that human infections with *A*. *phagocytophilum*, *B*. *burgdorferi*, SFG rickettsiae, and *F*. *tularensis* have been reported in various regions of China (*16**–**19*), the presence of these agents in rodents in the study areas suggests a potential threat to humans, and the public health role of these findings should be further investigated.

Although infectivity rates varied at different survey sites (Table, Appendix Table 1), we could not determine the geographic diversity of these agents in rodents. The number of rodents examined was limited; therefore, infectivity rates in the current study could be biased. In addition, because intensity of circulation of any vector-borne agent fluctuates dramatically throughout the year and from year to year, even at the same location (*20**,**21*), we could not justify comparing infectivity rates between different sites on the basis of unsynchronized single collections over a 3-year period. A randomized sampling scheme and further collection of rodents are required to clarify this issue. Unfortunately, we did not collect the ticks from captured rodents for additional testing of the tick-transmitted agents. This limitation prevented us from understanding vector potential.

In this study, *A*. *phagocytophilum* was detected only in eastern China (Table, Figure), where it coexists with the other 3 agents (Appendix Table 1). *A*. *phagocytophilum* detected in Heilongjiang, Jilin, and Zhejiang provinces were closely related to each other by 16S rRNA gene sequence analysis, but less related to other known strains in other countries. *B*. *burgdorferi* was detected in rodents from all 6 survey sites. As observed in a previous study (*9*), *B*. *garinii* was the dominant genospecies in mainland China, and the *B*. *valaisiana*–related group was present in southern China.

SFG rickettsiae, including ≈20 species of rickettsiae, can be transmitted to animals and humans not only by ticks but also by other arthropods such as infected lice, fleas, and mites (*10*). In this study, we amplified the *ompA* gene, which is present in most SFG rickettsiae (*10**,**22*). The overall infectivity rate for SFG rickettsiae was highest (9.1%) among the 4 agents tested (Appendix Table 1). Sequence analysis identified the *Rickettsia* sp. detected in Heilongjiang Province and Inner Mongolia Autonomous Region as a genotype of *R*. *sibirica*, which is known to cause Siberian tick typhus (*18*). However, we did not sequence PCR products amplified from rodents at other study sites because of a limited amount of samples. Although sequence analysis of the *ompA* gene fragment is not sufficient to identify the agent (*22*), it is commonly used to recognize tick-borne *Rickettsia* spp. in field surveys (*23*).

*F*. *tularensis* was found only in northern China, which verifies our belief that *F*. *tularensis* is present only north of 30°N latitude. In many disease-endemic areas, ticks are known to play a role in transmitting *F*. *tularensis* from animal hosts to humans, although other arthropods such as deer flies, fleas, mites, and mosquitoes are known to carry the bacterium. Sequence analysis showed that all *F*. *tularensis* detected in this study belong to the subspecies *holarctica*.

Interference of infections among *A*. *phagocytophilum*, *B*. *burgdorferi*, SFG rickettsiae, and *F*. *tularensis* in rodent hosts is not clear. Our findings indicate that infection with *A*. *phagocytophilum* does not intensify risk for transmission of the other 3 agents and vice versa. *B*. *burgdorferi* in rodents appears to increase risk for infection with *F*. *tularensis* but does not increase the possibility of infection with SFG rickettsiae or *A*. *phagocytophilum*. Further investigations are needed to demonstrate positive or negative interactions of the pathogens and to establish whether this interference is associated with the animal species.

Of 705 rodents tested in this study, 15 were infected with 2 agents and 3 were infected with 3 agents. These findings indicate that mixed natural foci of tick-borne agents are present at the study sites. Because *A*. *phagocytophilum*, *B*. *burgdorferi*, SFG rickettsiae, and *F*. *tularensis* were found in ticks collected in the study areas (*6**–**9**,**12**–**14*), it is not surprising that multiple agents were detected in rodents. Coexistence of multiple agents might be caused by a single bite of a tick infected with several agents or multiple bites of ticks infected with at least 1 agent. The presence of 4 pathogens in the study areas demonstrates the risk for multiple infections in humans, which may lead to variations and exacerbation of clinical signs (*1*). Therefore, differential diagnoses should be made for febrile patients with a history of tick bites in these areas, particularly when clinical signs are atypical for 1 disease or a related disease.

Among 23 rodent species trapped in this study, 21 were infected with >1 agent ( Appendix Table 2). Only 2 species (*Cricetulus migratourius* and *N*. *fulvescens*) were negative for all 4 agents. Which species is the main host of each agent remains unknown, because none of the agents are predominantly associated with 1 or a few related rodent species, regardless of their geographic origin. However, *A*. *phagocytophilum*, *B*. *burgdorferi*, SFG rickettsiae, and *F*. *tularensis* in various rodent species illustrate the potential roles of various rodents in maintaining these tick-borne agents. Systematic epidemiologic studies that investigate characteristics of natural foci and the role of small wild animals in transmission of these agents to humans are needed.

## Supplementary Material

Appendix Table 1 Co-infection rates for 4 tick-borne agents in rodents, People's Republic of China, 2004-2006*

Appendix Table 2 PCR results for 4 tick-borne agents in rodents, People's Republic of China, 2004-2006*
