# Supplementary material for: Tick-borne Agents in Rodents, China, 2004–2006
Source: Emerg Infect Dis. 2009 Dec;15(12):1904–8. doi: 10.3201/eid1512.081141 (PMC3044509; doi:10.3201/eid1512.081141)
Supplement: Appendix Table 1 — Co-infection rates for 4 tick-borne agents in rodents, People's Republic of China, 2004-2006* [file 08-1141_appT1-s1.pdf]

Publisher: CDC; Journal: Emerging Infectious Diseases
Article Type: Research; Volume: 15; Issue: 12; Year: 2009; Article ID: 08-1141
DOI: 10.3201/eid1512.081141; TOC Head: Research

Appendix Table 1. Co-infection rates for 4 tick-borne agents in rodents, People’s Republic of China, 2004–2006*

		No. (%) rodents positive												
Study site	No. rodents tested	*Anaplasma phagocytophilum* and *Borrelia burgdoferi*	*A. phagocytophilum* and SFG rickettsiae	*A. phagocytphilum* and *Francisella tularensis*	*B. burgdorferi* and SFG rickettsiae	*B. burgdorferi* and *F. tularensis*	SFG rickettsiae and *F. tularensis*	Co-infection	*A. phagocytophilum*, *B. burgdorferi*, and *F. tularensis*	*A phagocytophilum*, *B. burgdorferi*, and SFG rickettsiae	*A. phagocytophilum*, SFG rickettsiae, and *F. tularensis*	*B. burgdorferi*, SFG rickettsiae, and *F. tularensis*	Triple infection	Total no. (%)
Heilongjiang Province	64	0	0	0	0	0	0	0	0	1 (1.6)	0	0	1 (1.6)	1 (1.6)
Jilin Province	205	0	0	2 (1.0)	0	5 (2.4)	0	7 (3.4)	2 (1.0)	0	0	0	2 (1.0)	9 (4.4)
IMAR	148	0	0	0	2 (1.4)	0	0	2 (1.4)	0	0	0	0	0	2 (1.4)
XJAR	44	0	0	0	0	0	0	0	0	0	0	0	0	0
Zhejiang Province	216	1 (0.5)	1 (0.5)	0	3 (1.4)	0	0	5 (2.3)	0	0	0	0	0	5 (2.3)
Guizhou Province	28	0	0	0	1 (3.6)	0	0	1 (3.6)	0	0	0	0	0	1 (3.6)
Total	705	1 (0.1)	1 (0.1)	2 (0.3)	6 (0.9)	5 (0.7)	0	15 (2.1)	2 (0.3)	1 (0.1)	0	0	3 (0.4)	18 (2.6)

*SFG, spotted fever group; IMAR, Inner Mongolia Autonomous Region; XJAR, Xinjiang Autonomous Region.
