# Supplementary material for: Tick-borne Agents in Rodents, China, 2004–2006
Source: Emerg Infect Dis. 2009 Dec;15(12):1904–8. doi: 10.3201/eid1512.081141 (PMC3044509; doi:10.3201/eid1512.081141)
Supplement: Appendix Table 2 — PCR results for 4 tick-borne agents in rodents, People's Republic of China, 2004-2006* [file 08-1141_appT2-s2.pdf]

Publisher: CDC; Journal: Emerging Infectious Diseases
Article Type: Research; Volume: 15; Issue: 12; Year: 2009; Article ID: 08-1141
DOI: 10.3201/eid1512.081141; TOC Head: Research

Appendix Table 2. PCR results for 4 tick-borne agents in rodents, People's Republic of China, 2004–2006*

Species	No. (%) rodents tested	Study site, no. (%) rodents positive						No. (%) rodents positive				No. (%) co-infected
		Heilongjiang Province	Jilin Province	IMAR	XJAR	Zhejiang Province	Guizhou Province	*Anaplasma phagocytophilum*	*Borrelia burgdorferi*	SFG rickettsiae	*Francisella tularensis*	
Apodemus agrarius	142 (20.1)	13 (20.3)	75 (36.2)	44 (29.6)	0	10 (4.6)	0	14 (9.9)	4 (2.8)	27 (19.0)	14 (9.9)	4 (2.8)
A. peninsulae	74 (10.4)	13 (20.3)	56 (27.1)	5 (3.4)	0	0	0	4 (5.4)	9 (12.2)	1 (1.4)	7 (9.5)	3 (4.1)
A. sylvaticus	21 (3.0)	0	0	0	0	21 (9.7)	0	2 (9.5)	3 (14.3)	7 (33.3)	0	3 (14.3)
Cricetulus barabensis	11 (1.6)	0	0	10 (6.8)	0	1 (0.5)	0	0	1 (9.1)	0	0	0
C. migratourius	2 (0.3)	0	1 (0.5)	0	0	1 (0.5)	0	0	0	0	0	0
C. triton	5 (0.7)	0	5 (2.4)	0	0	0	0	0	1 (20.0)	0	1 (20.0)	1 (20.0)
Cricetulus sp.	39 (5.5)	0	17 (8.3)	0	5 (11.3)	17 (7.9)	0	2 (5.1)	0	0	2 (5.1)	0
Clethrionomys rufocanus	65 (9.2)	37 (57.8)	23 (11.1)	5 (3.4)	0	0	0	3 (4.6)	6 (9.2)	1 (1.5)	2 (3.1)	1 (1.5)
Clethrionomys rutilus	13 (1.8)	0	0	13 (8.8)	0	0	0	0	0	1 (7.7)	0	0
Meriones unguieulataus	15 (2.1)	0	0	0	15 (34.1)	0	0	0	0	1 (6.7)	1 (6.7)	0
Microtus maximowiczii	35 (4.9)	0	0	35 (23.7)	0	0	0	0	0	0	1 (2.9)	0
Mus pahari	7 (1.0)	0	0	0	0	0	7 (25.0)	0	0	1 (14.28)	0	0
Mus musculus	46 (6.5)	0	4 (1.9)	12 (8.1)	22 (50.0)	0	8 (28.6)	0	2 (4.7)	8 (18.6)	0	1 (1.3)
Niviventer confucianus	115 (16.2)	0	0	0	0	115 (53.2)	0	6 (5.2)	10 (8.7)	8 (7.0)	0	2 (1.7)
N. coxingi	4 (0.6)	0	0	0	0	4 (1.8)	0	1 (25)	1 (25)	0	0	0
N. fulvescens	2 (2.8)	0	0	0	0	2 (0.9)	0	0	0	0	0	0
Rattus losea	32 (0.5)	0	0	0	0	30 (13.9)	2 (7.1)	1 (3.1)	3 (9.4)	6 (18.8)	0	1 (3.1)
R. norvegicus	47 (6.6)	1 (1.6)	13 (6.2)	12 (8.1)	0	12 (5.6)	9 (32.2)	4 (8.5)	3 (6.4)	2 (4.3)	2 (4.3)	1(2.1)
Tamias sibiricu	18 (2.5)	0	11 (5.3)	7 (4.7)	0	0	0	1 (5.6)	2 (11.2)	0 (0)	5 (27.8)	1 (5.6)
NI	6 (0.9)	0	0	5 (3.4)	0	1 (0.5)	0	0	2 (33)	1 (17)	0	0
Others	6 (0.9)	0	0	0	2 (4.6)	2 (0.9)	2 (7.1)	1 (10.0)	0	0	0	0
Total	705 (100.0)	64 (100.0)	205 (100.0)	148 (100.0)	44 (100.0)	216 (100.0)	28 (100.0)	39 (5.5)	47 (6.7)	64 (9.1)	35 (5.0)	18 (2.6)

*SFG, spotted fever group; IMAR, Inner Mongolia Autonomous Region; XJAR, Xinjiang Autonomous Region; NI, not identified.
